# Practical application of hybrid effectiveness–implementation studies for intervention research

**DOI:** 10.1093/ije/dyaf039

**Published:** 2025-05-05

**Authors:** Margaret A Handley, Leah D Murphy, Elizabeth B Sherwin, Starley B Shade

**Affiliations:** Department of Epidemiology and Biostatistics, University of California San Francisco, San Francisco, CA, United States; Partnerships for Research in Implementation Science for Equity (PRISE) Center, University of California San Francisco, San Francisco, CA, United States; Institute for Global Health Sciences, University of California San Francisco, San Francisco, CA, United States; Partnerships for Research in Implementation Science for Equity (PRISE) Center, University of California San Francisco, San Francisco, CA, United States; Division of Pulmonary and Critical Care Medicine, School of Medicine, University of California, San Francisco, CA, United States; Department of Epidemiology and Biostatistics, University of California San Francisco, San Francisco, CA, United States; Partnerships for Research in Implementation Science for Equity (PRISE) Center, University of California San Francisco, San Francisco, CA, United States; Department of Epidemiology and Biostatistics, University of California San Francisco, San Francisco, CA, United States; Partnerships for Research in Implementation Science for Equity (PRISE) Center, University of California San Francisco, San Francisco, CA, United States; Institute for Global Health Sciences, University of California San Francisco, San Francisco, CA, United States

**Keywords:** hybrid studies, implementation science, intervention research

## Abstract

Hybrid effectiveness–implementation studies have emerged to address design challenges that researchers face when assessing evidence-based intervention implementation in real-world settings. Hybrid studies maximize the usefulness of effectiveness studies by allowing both effectiveness and implementation research questions to be included in the same study, regardless of the research design (observational, individually randomized trials, cluster-randomized trials, preference designs, adaptive designs, etc). Hybrid studies utilize implementation science methods to facilitate integration of research findings into public health programming, health-care practice, and community and policy settings. In this article, we describe the three types of hybrid studies, which exist on a continuum depending on the emphasis placed on the effectiveness vs the implementation research questions. We discuss common implementation outcomes with examples of how the stage (or phase) of implementation can influence selection of implementation outcomes. We then summarize hybrid study examples from recent literature and present practical considerations when designing a hybrid study. Finally, we outline examples of implementation-focused research questions to consider asking by phase of implementation and discuss related sampling approaches.

Key Messages

- Hybrid studies can improve the usefulness of effectiveness studies by answering both effectiveness and implementation questions.
- Hybrid studies often focus on exploring factors affecting the implementation of interventions in different settings.
- Considering the phase of implementation can influence the choice of implementation outcomes.
- Focusing on sampling for implementation outcomes during the early study planning can simplify data collection and conserve resources.

## Introduction and overview of hybrid studies

Intervention-focused researchers face many design challenges when implementing interventions in real-world settings. Researchers, including epidemiologists, can use implementation science methods to translate evidence-based interventions or programs into new settings and explore factors affecting implementation while concurrently evaluating intervention effectiveness. Implementation science incorporates a range of methods to promote the integration of research findings and evidence into policy and practice. It employs many different research designs (e.g. observational, individually randomized trials, cluster-randomized trials, preference trials, adaptive designs, etc) to assess not only the effectiveness of interventions in the “real world” but also how to implement interventions to best scale them, e.g. to optimize uptake or to deliver them at the lowest costs. In this context, hybrid effectiveness–implementation studies, often referred to as ‘hybrid studies’, have emerged to allow for both effectiveness and implementation research to be included in the same study, regardless of the research design [1–3]. Hybrid studies have at least one effectiveness and one implementation research question. Hybrid studies often embed implementation research questions *a priori*, but they can also add a separate evaluation after intervention implementation and explore implementation outcomes associated with the intervention (e.g. acceptability, feasibility, adoption, etc).

There are generally three types of hybrid effectiveness–implementation studies, each with many different approaches such that hybrid studies can best be thought of as spanning a wide continuum of approaches. In this article, we provide: (1) a summary and examples of the three broad types of hybrid studies; (2) common implementation outcomes frequently used in hybrid studies (summarized in Table 1) and how they can be applied to a range of research designs also evaluating effectiveness; (3) key considerations of hybrid study planning including sampling considerations; (4) examples of recent hybrid studies with associated effectiveness and implementation research questions and data collected for each type of outcome (see Table 2); and (5) considerations for sampling for different areas of focus for hybrid studies (see Table 3).

**Table 1.** Widely used implementation outcomes in hybrid studies with examples of relevant research questions

Implementation outcomes	Definition	Related framework	Level of analysis	Program focused research questions	Population focused research questions	Equity focused research questions
Implementation outcomes for consideration in early phases of implementation (Exploration and Preparation Phases^a^)						
Acceptability	‘The perception among stakeholders that a given treatment, service, practice, or innovation is agreeable, palatable, or satisfactory’	Proctor implementation outcomes	Individual provider and/or individual participant	Are all elements of the intervention equally acceptable, or is there important variation in acceptability across sites?	Are levels of acceptability similar among sub-populations (e.g. for men and women and across age groups)?	Is the intervention equally acceptable to specific target groups that are intended to receive it, as to others across the program?
Appropriateness	‘The perceived fit, relevance, or compatibility of the treatment or innovation or a given practice setting, provider, or consumer; and/or the perceived fit of the innovation to address a particular issue or problem’	Proctor implementation outcomes	Individual provider and/or individual participant Organization or setting	Do staff delivering the intervention find it to fit well within their scope of practice and goals for addressing the needs it is intended to fill?	Do sub-populations receiving the intervention find it to meet their needs and not raise any concerns?	Are there any identified concerns that intervention elements will not be well-received by some sub-groups?
Feasibility	‘The extent to which a new treatment or innovation can be successfully used or carried out within a given setting’	Proctor implementation outcomes	Individual provider Organization or setting	Does the intervention present any challenges that interfere with implementing it?	Are some potential participants not offered the intervention? And if so, for what types of reasons and what factors might overcome barriers?	Are there any specific groups of potential participants that are not offered the intervention? If so, for what reasons and what factors might overcome barriers?
Implementation outcomes for consideration in the middle phase of implementation (Implementation Phase^a^)						
Adoption	‘The action to try to employ a new treatment or innovation–also referred to as “uptake”’	RE-AIM; proctor implementation outcomes	Organization or setting	Are sites that are offered the intervention equally likely to implement it (e.g., have similar proportions of uptake)?	Are there site-specific characteristics that prevent (or enable) some sites to offer the intervention, compared to sites with different characteristics?	Are sites with greater proportions of sub-groups that face barriers to accessing the intervention equally likely to have intervention uptake?
Availability	‘The supply of a new treatment or innovation at any one time’	Pilar *et al.* 2022 [13]	Individual provider Organization or setting Systems or policy	Do sites offering the intervention have uninterrupted access to the new treatment or innovation?	Are there sub-populations that may face increased challenges in accessing a steady supply of this new treatment or innovation? If so, for what reasons and what factors might overcome barriers?	Is the new treatment or innovation available to all sub-groups that could benefit from it?
Fidelity	‘The degree to which a treatment or innovation was implemented as it was prescribed in the original protocol or as was intended by the program developers’	Proctor implementation outcomes	Individual provider	Do program staff provide the same intervention components across sites, or are there some situations where the intervention is modified, and if so, why?	Are the same intervention components, dose and quality offered to all potential participants across all site and service settings?	Are there specific groups that are not receiving all intervention components, for example a reduced dose or reduced quality of the intervention?
Penetration	‘The integration of a treatment or innovation within a service setting and its subsystems’	Proctor implementation outcomes	Organization or setting	To what extend do staff delivering the intervention find it to be well-integrated into their workflows?	Is there variation in the degree to which sites have integrated the intervention into their service settings by target sub-population (e.g. rural vs urban and across age groups)?	Are there specific groups who are eligible for the intervention, but are not being reached and/or are not utilizing the intervention?
Reach	‘The proportion of the target population that participated in the intervention’	RE-AIM^b^	Individual participant	Do intervention participant rates vary across sites?	Is there variation in participation rates among key sub-populations?	Are there specific sub-groups with lower participation rates? If so, what are the reasons and how can barriers be addressed to increase participation among the sub-group?
Implementation outcomes for consideration in the advanced phase of implementation (Sustainment Phase^a^)						
Cost	‘The cost impact of an implementation effort’	Proctor implementation outcomes	Individual provider Organization or setting	What are the costs associated with the intervention, including the variation in costs across sites?	Do certain sub-populations require more intensive/costly intervention delivery services?	Are some sites experiencing unanticipated costs that mean that some sub-groups are not receiving the intervention?
Scale-up	‘Deliberate efforts to increase the impact of successfully testing health innovations to benefit more people and to foster policy and program development on a lasting basis’	Pilar *et al.* 2022 [13]	Organization or setting Systems or policy	Are there systems, infrastructure, or technological challenges outside the full control of the site that may inhibit wide-spread implementation of the intervention?	Are there health systems, infrastructure or technological challenges inhibiting wide-spread implementation of the intervention among certain sub-populations?	Are there structural, environmental or systems barriers that enable some groups to benefit from the intervention, while other groups do not benefit or for which the benefit is greatly reduced?
Sustainability/maintenance	‘The extent to which a treatment or innovation is maintained or institutionalized within a service setting’s ongoing, stable operation’	RE-AIM; proctor implementation outcomes	Organization or setting	Are sites still offering the intervention following the departure of research-associated teams?	Are their site-specific characteristics that prevent (or enable) some sites from offering the intervention to target sub-populations on a long-term basis?	Are there competing needs or other barriers that result in some groups not receiving the intervention on a long-term basis?
Implementation outcomes for consideration in all phases of implementation						
Health Equity	‘Fair access to a treatment or innovation without avoidable or remediable differences among groups of people’	Pilar *et al.* 2022 [13]	Individual provider and/or individual participant Organization or setting Systems or policy	Is the intervention offered to sites that may face challenges in implementation, but also may be able to have the greatest impact (e.g. those with more intensive service delivery needs or other specific challenges)?	Are adequate resources and technical assistance provided across sites to ensure all sub-populations have access to the intervention?	Is the intervention unacceptable to any of the sub-groups that are intended to receive it? If so, which elements and for what reasons?

a Implementation phases are from the Exploration, Preparation, Implementation, Sustainment (EPIS) implementation science framework [14].

b Reach, Effectiveness, Adoption, Implementation, and Maintenance (RE-AIM) framework.

**Table 2.** Examples of hybrid studies showcasing a range of intervention evaluation study designs^a^

Intervention Description/study overview	Research question: Effectiveness	Research question(s): Implementation	Study design	Data collected: Effectiveness outcomes	Data collected: Implementation outcomes	Key implementation outcomes assessed	Implementation science frameworks used (if applicable)
Hybrid 1							
‘Khanya’, a task-shared, peer-delivered behavioral intervention to improve ART adherence and reduce alcohol and other drug use in HIV care in South Africa (*N* = 61) [15]	Is ‘Khanya’ associated with improvements in ART adherence over three months and reduced alcohol and other drug use over six months vs enhanced treatment as usual?	What is the feasibility, acceptability, appropriateness, and fidelity of the peer-delivered intervention (‘Khanya’)?	Individual RCT	Quantitative: ART adherence (monitoring device) and alcohol and drug use (biomarker and self-report)	Quantitative (session attendance and participant questionnaire)	Feasibility, acceptability, appropriateness, and fidelity	Proctor [11]
Exercise intervention for firefighters (on-site supervised exercise (*n* = 86) or telehealth exercise (*n* = 95)) compared to control group (*n* = 83) [6]	Does worksite exercise reduce lost work time related to low back pain in firefighters compared to a control group?	What are the barriers and facilitators to implementation of long-term worksite exercise programs among firefighters?	Cluster RCT	Quantitative: lost work time related to back pain (participant questionnaires, administrative documents)	Quantitative (exercise logs, participant questionnaires) and Qualitative (focus groups and observations)	Adherence, barriers, facilitators	
‘Peer Activate’, a peer recovery specialist-delivered behavioral activation approach to improve methadone retention for low-income, largely racial/ethnic minoritized individuals in opioid use disorder treatment (*n* = 37) compared to comparison cohort (*n* = 119) [17]	Does ‘Peer Activate’ improve methadone retention for individuals in opioid use disorder treatment compared to a comparison cohort?	What is the feasibility, acceptability, and fidelity of ‘Peer Activate’?	Pilot cohort study	Quantitative: Retention data (methadone dosing data)	Quantitative (session attendance, participant questionnaire) and Qualitative (audio-recorded sessions)	Feasibility, acceptability, fidelity	Proctor [11]
StayWell at Home (StayWell), a 60-day text messaging program to help Latine adults cope with depressive and anxiety symptoms during the pandemic (*n* = 109) compared to non-Latine white users (*n* = 289) [7]	Is the effectiveness of StayWell in improving anxiety and depression symptoms consistent among Latine and non-Latine white users during the pandemic?	What is the reach, adoption, implementation, and maintenance of StayWell in Latine compared to non-Latine white users? What are the barriers and facilitators experienced by users?	Comparative effectiveness	Quantitative: anxiety and depressive symptoms (validated scales)	Quantitative (user surveys) and qualitative (open-ended questions on surveys)	Reach, adoption, maintenance, barriers, and facilitators	RE-AIM [12]
Hybrid 2							
A new care pathway combining midwifery continuity of care with a specialist obstetric clinic (POPPIE) compared to standard maternity care for women at risk of preterm birth in London (*N* = 334) [18, 19] Patients receiving the POPPIE intervention receive care from the same midwife throughout pregnancy and postpartum, whereas standard of care patients receive care from different midwifes	Can POPPIE improve a composite outcome of timely and appropriate interventions provided for the prevention and/or management of preterm labor and birth compared to standard of care?	What is the appropriateness, adoption, feasibility, acceptability, fidelity, penetration, and sustainability of POPPIE?	Individual RCT	Quantitative: clinical and process outcomes (e.g. total number of visits, total inpatient nights, total number of referrals)	Quantitative (meeting records, key documents, participant surveys) and Qualitative (participant interviews)	Appropriateness, adoption, feasibility, acceptability, fidelity, penetration, sustainability	Proctor [11] and CFIR [20]
The addition of Implementation and Sustainment Facilitation (ISF) strategy/component to the Addiction Technology Transfer Center (ATTC) intervention for integrating motivational interviewing-based brief intervention (MIBI) for substance use disorders within HIV community-based organizations (*n* = 39 organizations and *n* = 824 client participants). [21] The ISF intervention includes seven implementation strategies/components not included in the ATTC strategy: external facilitation, tools for quality improvement, implementation team meetings, identifying and preparing champions, assessing readiness and barriers, local consensus discussions, and conducting cyclical tests of change	Does the standard ATTC strategy + ISF strategy reduce client substance use-related problems, and engagement in risky behaviors and increase substance use treatment and medication adherence compared to ATTC alone?	Does the ISF strategy improve staff-level consistency and quality of MIBI implementation?	Cluster RCT	Quantitative(days of primary substance use, number of substance-related problems, times engaging in risky behaviors, days of substance use treatment, and days of medication non-adherence)	Quantitative (staff-level measures developed using number of MIBIs implemented and MIBI proficiency scores)	Consistency, quality, sustainment	CFIR [20]
Music-with-movement intervention for people with dementia and their caregivers (*N* = 100 dyads) [22]. The study includes 18 implementation strategies/components related to assessing readiness and providing feedback, team meetings, outreach, education, engagement, and local consensus discussions	Does the music-with-movement intervention improve the psychosocial well-being of people with dementia and their caregivers, and the quality of the dyadic relationship, compared to a wait-list control group?	What is the acceptability, appropriateness, feasibility, adoption, and sustainability of the implementation strategies used to promote adoption of the music-with-movement intervention?	Cluster RCT (with wait-list control)	Quantitative: anxiety and depressive symptoms, stress, caregiver relationship (validated scales)	Quantitative (measures of reach and adoption) and Qualitative (focus groups)	Acceptability, appropriateness, feasibility, adoption, sustainability, facilitators, barriers	Expert Recommendations for Implementing Change (ERIC) [5] and CFIR [20]
‘mySTEPS’, a psychoeducational intervention for improving heart-healthy behaviors for women of low SES (*N* = 42) [23]. Participants receive interactive, educational sessions and one-on-one nurse coaching sessions. mySTEPS intervention components include: developing clinic partnerships, tailoring intervention to the target population, nurse education, ongoing implementation monitoring and evaluation, providing services in neighborhood community settings, and providing free onsite childcare	Does the ‘mySTEPS’ intervention improve the health and health behaviors of women of low SES compared to before the program?	What is the reach, fidelity of delivery and receipt, acceptability, and appropriateness of the ‘mySTEPS’ intervention adapted for women of low SES?	Pre-post	Quantitative: perceived stress, physical symptoms, physical activity, dietary intake (pre and post surveys)	Quantitative (attendance, adherence, participant surveys) and Qualitative (open-ended survey questions)	Reach, fidelity, acceptability, and appropriateness	
Medication therapy management across Medicaid’s pilot program in medication therapy management in Tennessee (TennCare), a service offered by pharmacists to ensure a patient’s medication regimen is individualized to include the safest and most effective medications (*N* = 24 providers) [24, 25]. The study includes seven implementation components related to planning, education, finance, data systems, and quality management	What is the effectiveness of the TennCare pilot program on medication adherence, healthcare utilization, and quality and cost of care?	At the organizational and individual level, what is the adoption, feasibility, acceptability and appropriateness of a multi-faceted implementation strategy to support the TennCare pilot? What are the contextual factors associated with program implementation?	Pilot cohort study	Quantitative: medication adherence, healthcare utilization, healthcare cost, quality of care	Quantitative (billing claims to measure adoption and adherence, provider surveys) and qualitative (interviews with providers)	Adoption, feasibility, acceptability, appropriateness, barriers, facilitators	CFIR [20]
Model to improve selective screening for Gestational Diabetes Mellitus (GDM) in pregnant women (*N* = 1073) [26] compared to the formerly used screening approach (testing women with one or more risk factors for GDM)	How does an externally validated prognostic model compare to the formerly used selective screening approach to identify GDM?	What is the adoption, acceptability, appropriateness, feasibility, fidelity, penetration, and sustainability of an externally validated prognostic model to improve selective screening for GDM?	Cohort with modeling	Quantitative: model performance and clinical outcomes (from medical records)	Quantitative (validated instruments and process indicators collected from both obstetric healthcare professionals and pregnant women)	Adoption, acceptability, appropriateness, feasibility, fidelity, penetration, sustainability	Proctor [11]
Telehealth model of primary care mental health integration (tele-PCMHI) at satellite Veterans Health Administration clinics (*N* = 6 sites) [8, 9]. As part of the study, implementation facilitators develop an implementation plan, support problem solving and provide technical support, and revise and adapt programs based on feedback	Does the tele-PCMHI model improve clinical health outcomes and referral management compared to usual care?	What is the provider reach into the patient population, adoption by providers, and implementation and innovation fidelity of the tele-PCMHI model?	Stepped wedge	Quantitative clinical outcomes and mental health referrals (from medical records)	Quantitative (use of tele-PCMHI) and Qualitative (interviews and field notes)	Reach, adoption, fidelity	Integrated-Promoting Action on Research Implementation in Health Services (i-PARIHS) [27]
Hybrid 3							
‘Rural School Support Strategies (RS3)’, a bundle of supports for Positive Behavioral Interventions and Supports (PBIS) implementation in rural schools in Idaho (N = 40 schools) [10, 28] The support bundle includes remote and virtual technical assistance, monthly virtual learning sessions, and access to educational materials and resources	Do student outcomes differ for students attending schools randomized to RS3 as compared to students at schools in the control group?	Does standard training plus the supports of RS3 improve PBIS implementation fidelity relative to a training-only control group? What are the mediators of the effectiveness of RS3 on implementation fidelity? What is the feasibility, acceptability, appropriateness, and costs of RS3?	Cluster RCT	Quantitative: student disciplinary incidents, academic outcomes, climate surveys	Quantitative (surveys) and Qualitative (interviews)	Fidelity, acceptability, appropriateness, feasibility, cost	Quality implementation framework [29]
A multi-component training package for the Collaborative Model for Promoting Competence and Success (COMPASS), a multilevel consultation and coaching intervention for improved educational outcomes of students with autism (*N* = 105 students, parents, teachers, and consultant-trainees) [30]. In the COMPASS intervention a consultant facilitates a parent–teacher consultation in which parent and teacher develop learning goals and a personalized plan for the child, as well as follow-up coaching sessions with the teacher	What is the impact of the training package on consultant-trainee knowledge of evidence-based practices and attitudes?	Can we develop a training package for autism consultants that results in acceptable levels of implementation outcomes (appropriateness; feasibility; fidelity)? How much follow-up/feedback was necessary to achieve acceptable implementation outcomes? Would parents and teachers report COMPASS when delivered by school consultants as acceptable, appropriate, and feasible?	Cohort study	Quantitative: knowledge scale (consultant–trainee surveys)	Quantitative (surveys of parents, teachers, and consultant–trainees)	Acceptability, fidelity, appropriateness, feasibility	

a Table adapted from Curran *et al.* [1].

b ART, antiretroviral therapy; ATTC, Addiction Technology Transfer Center; CFIR, Consolidated Framework for Implementation Research; COMPASS, Collaborative Model for Promoting Competence and Success; GDM, Gestational Diabetes Mellitus; i-PARIHS, Integrated Promoting Action on Research Implementation in Health Services framework; ISF, Implementation and Sustainment Facilitation; MIBI, motivational interviewing-based brief intervention; PBIS, Positive Behavioral Interventions and Supports; PCMHI, primary care mental health integration; POPPIE, pilot study of midwifery practice in preterm birth including women’s experiences; RCT, randomized controlled trial; RE-AIM, Reach, Effectiveness, Adoption, Implementation, and Maintenance framework; RS3, Rural School Support Strategies; TennCareMTM, medication therapy management across Medicaid’s pilot program in medication therapy management in Tennessee.

**Table 3.** Sampling considerations for implementation outcomes in hybrid studies

Possible implementation outcome questions	Sample-related questions
How feasible and acceptable are the intervention components, and the intervention overall, to those who are the implementors and those who are intended to receive the program? What does variation look like in acceptability/uptake among participants? Are there modifiable barriers that might be addressed for those participants with lower levels of engagement?	Sample questions Do you want to explore participant perspectives that reflect variation in acceptability measures? Or do you want to understand the acceptability among different types of providers or community members based on characteristics (rural, urban, age, gender)? Do you want to examine how feasible the intervention was for different groups of implementors?
What is the reach of the intervention into the target population, compared with standard of care/routine practice? What does variation look like in fidelity and adoption among sites delivering the intervention?	Targeted sample questions Do you want to explore participant perspectives that reflect variation in reach for each intervention? Do you want to examine what factors affected levels of fidelity or adoption of the interventions for different groups of implementors? Do you want to explore how different groups are engaging in the intervention (e.g., by demographic, health status, barriers, enablers)?
What is the comparative cost of the different forms of an intervention (e.g. delivery, intensity), comparison of different interventions, or costs of de-implementing/swapping one practice for another? What does variation look like in acceptability, uptake, fidelity, and costs? How are different groups engaging in each type of intervention? Are there modifiable barriers for some groups or those with lower levels of engagement?	Targeted sample questions Do you want to explore participant perspectives that reflect variation in staff resource use for each intervention to identify those that are most acceptable to implementors? Do you want to determine what factors (patient characteristics, staffing models) affect variation, sustainability and costs for each intervention? Do you want to identify which community members are most engaged with each of the interventions over time?

Hybrid type 1 studies focus primarily on establishing the effectiveness of an intervention and explore the “implementability” of an intervention [2], for which a range of implementation outcomes may be relevant to the study. The term intervention in this article refers to single or multiple policies, practices, or products intended to improve health behaviors, health outcomes, or health-related environments. Example interventions include taxation of cigarettes, integration of trauma screening into HIV care and treatment, peer delivery of substance use education, and use of a text platform to enhance mental health counseling among adolescents. Implementers employ implementation strategies–actions to enhance adoption, implementation, or sustainment of an intervention. Example implementation strategies include assessing readiness and identifying potential barriers and facilitators, developing a formal implementation blueprint, identifying early adopters, making training more dynamic, and providing ongoing supervision [4]. A taxonomy, based on the Expert Recommendations for Implementing Change (ERIC) study, summarizes widely used implementation strategies [5].

Hybrid type 1 studies are best suited when there is early evidence of efficacy, but it is still unclear if an intervention is effective either overall or in a particular population. The main research question examines whether the intervention is effective. Secondary implementation research questions are included and may focus on better understanding the context for implementation, often using a combination of qualitative and quantitative data collection or mixed methods approaches. Examples from Table 2 of implementation outcomes for hybrid type 1 studies which combine quantitative and qualitative assessment include: how to understand barriers and enablers to adoption of an exercise program for firefighters to reduce low back pain (focus groups and observations) [6] and determining to what extent a mental health support program has similar uptake or reach when delivered to Latine adults vs other groups (open-ended survey questions) [7]. The hybrid type 1 study is usually powered to detect a difference for the effectiveness outcome, reflected in sample size calculations. However, hybrid type 1 studies may also assess implementation outcomes.

Hybrid type 2 studies have a dual focus on effectiveness and implementation research question(s). Evaluation of effectiveness in hybrid type 2 studies usually involves comparing those who do and those who do not receive the intervention(s) in the full sample, but for evaluation of implementation outcomes there may be a sampled sub-group to explore factors affecting implementation in more depth, often with the use of qualitative methods. Hybrid type 2 studies are well-suited to situations which focus on measuring the impact of a particular intervention strategy, e.g. 1:1 coaching or providing reminders, and at the same time, evaluating changes in health indicators.

A hybrid type 2 study may involve randomization, either at the individual or site level to evaluate the primary intervention outcome (e.g. obesity, air pollution), while also including a single-arm, pilot study of an intervention strategy (e.g. online training of all providers/sites). This example would be considered a hybrid type 2 study as there are dual goals of assessing the impact of both clinical/prevention interventions and implementation strategies. Other hybrid type 2 studies, such as those intended for feasibility/pilot data collection, would not be powered for a specific outcome, by the very nature of being a pilot study. Examples of hybrid type 2 studies which involve both quantitative and qualitative approaches are described in Table 2 representing a range in data collection approaches (focus groups, in-depth interviews, observations, and open-ended survey questions) [8, 9]. Sample size estimation for a hybrid type 2 study can focus on detecting differences for both the implementation and effectiveness outcomes or either type of outcome.

Hybrid type 3 studies focus on assessing the implementation of interventions to determine which is best suited for the setting and situation. The effectiveness of the program on health outcomes is viewed in hybrid type 3 studies as less essential to measure, as the evidence for beneficial health impacts have previously been established. Such a situation may arise when an intervention has well established effectiveness in a specific setting, yet critical questions arise as to how to improve the program or reduce the overall costs by offering a different delivery strategy. As such, a hybrid type 3 study may compare different intervention delivery strategies [10], but there will also be measurement of the health impacts from the program.

## Types of implementation outcomes

While epidemiologists are familiar with effectiveness outcomes (e.g. health behaviors, health outcomes, or health-related environments), many are not familiar with implementation outcomes. Implementation outcomes (i.e. outcomes that assess aspects of implementation) are essential for evaluating efforts to implement an evidence-based intervention in real-world settings. An evidence-based intervention is unlikely to be effective in routine practice settings if it is not implemented well [11]. Within the field of implementation science, there is not currently a one-size-fits all approach to identifying implementation outcomes to measure and the level at which they will be measured (i.e. individual community member, client/provider, clinic, organization, region). Several commonly measured implementation outcomes are defined in Table 1; the majority are based on the Implementation Outcomes Framework (acceptability, adoption, appropriateness, costs, feasibility, fidelity, penetration, and sustainability) [11] and the RE-AIM Framework (reach, adoption, maintenance) [12]. Additional implementation-related outcomes (availability, health equity, and scale-up) are also widely used [13].

While implementation outcomes can be measured throughout implementation, some implementation outcomes may be more relevant at certain stages or phases in the implementation process. The EPIS (Exploration, Preparation, Implementation, and Sustainment) implementation science framework maps the implementation process across phases of implementation [14] and provides a useful way to organize commonly used implementation outcomes by stage of the implementation process (Table 1). For example, it is common to explore acceptability, appropriateness, and feasibility in the earlier stages of research, e.g during the pre-implementation phase, to understand if the proposed intervention is perceived as relevant and practical among key stakeholders (i.e. providers, patients, program administrators). Adoption, availability, fidelity, penetration, and reach are typically measured in the middle phase of research, during the active implementation phase. Cost, scale-up, and sustainability/maintenance are often measured in the later phase of research, during the sustainment phase of implementation. However, any of these outcomes can be measured in earlier stages of implementation depending on the research question. Equity can also be useful to measure throughout implementation. For example, considering equity in acceptability or reach in the pre-implementation phase can help ensure the proposed intervention can impact a wide range of participants.

Table 1 offers examples of the types of questions researchers can ask to explore implementation outcomes within different contextual settings and for select sub-populations. They are organized to allow for different types of questions that may be related to program-focused, population-focused, and equity-focused research. The program-focused research questions illustrate the types of questions particularly relevant to explore implementation outcomes within a specific program setting (e.g. reach across sites, fidelity vs adaptation of program/intervention). The population-focused questions provide examples of research questions to assess implementation outcomes among key sub-populations (i.e. rural vs urban and age groups). The equity-focused questions offer examples of research questions to assess variation in implementation settings (i.e. public vs private clinic settings) or variation in other contextual factors, such as geography or language, that are of particular importance when exploring implementation outcomes among sub-groups experiencing health inequities.

Table 2 presents recent examples of hybrid studies covering a range of epidemiologic research designs including cohort studies and individual and cluster-randomized controlled trials (RCTs). As shown in the second and third columns of Table 2, researchers can ask the same types of questions regardless of hybrid study type. The key difference is that type 1 studies emphasize the effectiveness question, Type 3 studies emphasize the implementation question, and Type 2 studies emphasize both types of questions. For example, Table 2 includes two RCTs studying interventions for medication adherence among adults accessing HIV/AIDS care [15]. Type 1 study’s primary question is about the effectiveness of the intervention regarding medication adherence [15], whereas Type 3 trial focuses on studying the optimal approach for receipt of medication.

The implementation research question should guide the implementation outcomes measured and researchers often use implementation science frameworks to organize implementation questions and outcomes. As described by Nilsen, theories, models, and frameworks have specific functions (e.g. frameworks that help explain the intervention and barriers and enablers in context/determinants frameworks; those that guide implementation; and those that focus on evaluation) [16]. Depending on the goals of the study, one might use different frameworks to select outcomes of interest (e.g. barriers/facilitators to implementation of something [via a determinant framework] vs relative performance of strategies on implementation outcomes such as reach, adoption or fidelity using an evaluation framework). For example, in Table 2, a hybrid type 1 study of a text messaging program (StayWell) [7] to help Latine adults cope with depressive and anxiety symptoms used the RE-AIM framework [12], an evaluation framework, to organize their implementation outcomes.

## Practical considerations: choosing a hybrid study and approaches to sampling

**Figure 1.**
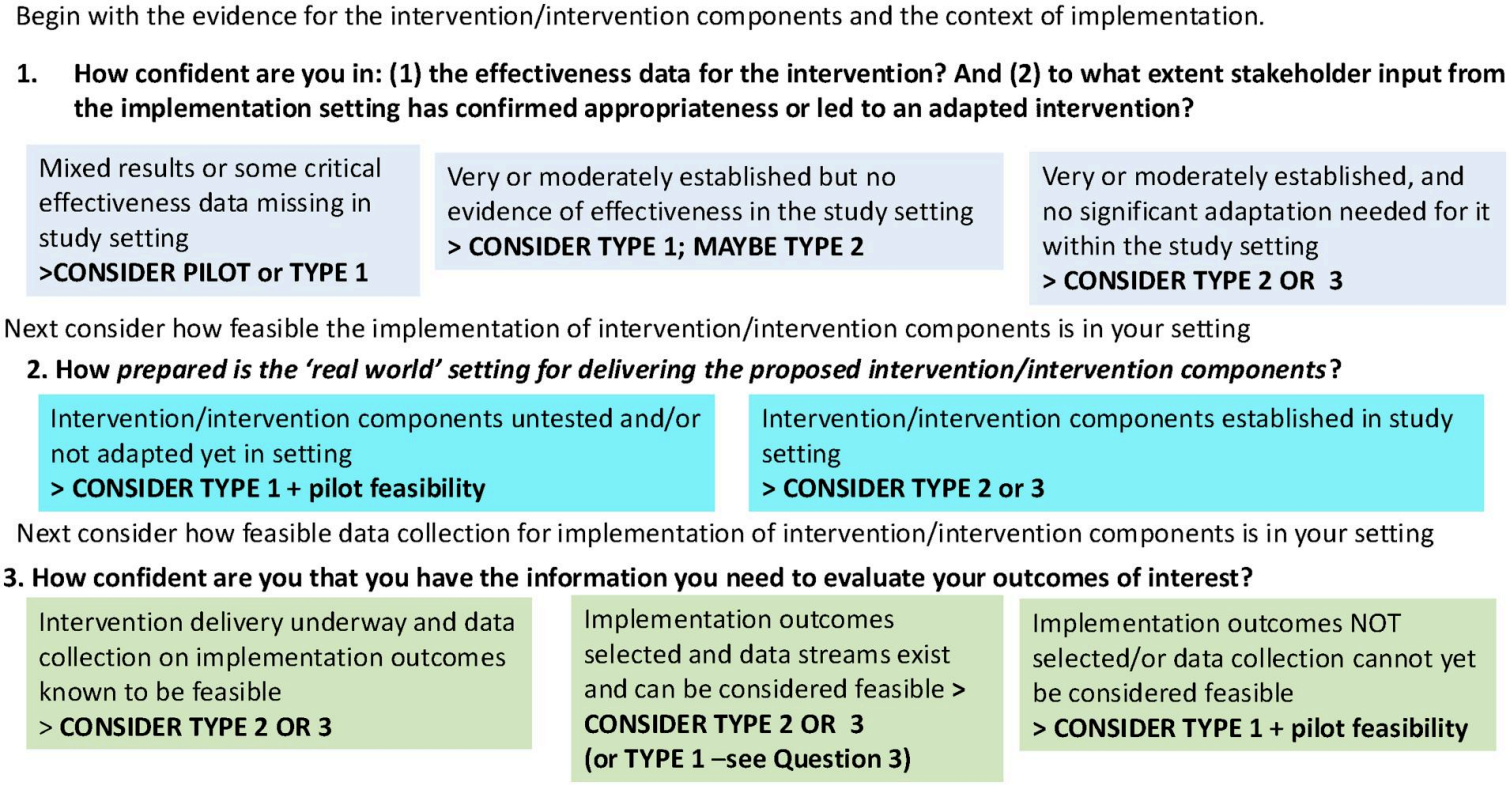
Steps to consider which hybrid study may be best suited for the intervention study being planned. Figure adapted from Curran *et al*. [3].

There are several ways to decide which hybrid study is best suited to the research setting and questions. In Fig. 1, we outline example questions that can guide hybrid study selection. There are two essential questions to address at the outset. First, how confident you are in the evidence-base for the intervention’s effectiveness and related, do stakeholders also agree that the evidence for the specific intervention applies to their setting?’ If the evidence-base is not yet established in the setting/population you plan for the study, a hybrid type 1 study would be most suitable. If, however, there is moderate evidence in the setting and for the population of effectiveness, you are more likely to consider a hybrid type 2 or hybrid type 3 study.

The second consideration relates to how prepared the setting is for delivery. In the absence of information about how well the proposed intervention components can be integrated into existing sites, then a pilot study or hybrid type 1 study makes more sense. If there are clear pathways to study implementation, then a hybrid type 2 or hybrid type 3 study may be considered. The third consideration concerns how feasible it would be to evaluate the intervention by collecting appropriate outcome data (for effectiveness and implementation outcomes). If an intervention is already underway or data collection mechanisms for the selected implementation outcomes are in place, either a hybrid type 2 or hybrid type 3 study can be considered. If there is no information about how to collect implementation outcomes, conducting a small pilot study to establish data collection procedures, or a hybrid type 1 study, would be most appropriate.

Focusing on sampling for implementation outcomes during the early study planning can simplify data collection and conserve resources. In Table 3, we present examples of research questions to consider for implementation outcome questions with related sampling considerations. With an early pre-implementation phase implementation study, there may be an emphasis on determining the acceptability of different intervention strategies, such as training, nudging, coaching, etc. Implementation-related questions might include: ‘How acceptable is the intervention to staff delivering it?’ And a related sampling frame question might be: ‘Would the research question be best answered by sampling on a specific level of acceptability obtained during a survey, by taking a random sample of all staff, or a stratified sample based on job title or length of time in the position?’ Specific sampling questions might then focus on: ‘Do you want to identify barriers to acceptability among staff with the lowest levels of reported acceptability compared to enablers among staff with high levels of reported acceptability?’ Examples are similarly provided for middle/later implementation phases. For any study, it is important to think about if you want to sample based on specific outcomes (e.g. high or low acceptability, feasibility, reach, engagement, cost, sustainability, etc), certain sub-groups (e.g. job classification or demographics), or simply conduct a random sample.

Hybrid studies frequently incorporate qualitative methods such as interviews and focus groups, to explore specific research questions in more depth. The addition of qualitative methods to hybrid studies can enhance interpretation of results by shedding light on questions related to the “why” or “how” of the implementation process. For example, the hybrid type 2 ‘mySTEPS’ study, which is a psychoeducational intervention for improving heart-healthy behaviors [23], interviews were employed to study implementation context. Other implementation outcomes lend themselves to quantitative data; ‘mySTEPS’ [23] used billing claims to measure adoption of the program.

## Summary

In this article, we describe common examples of hybrid effectiveness–implementation studies with a focus on: describing widely used implementation outcomes; linkages between types of hybrid studies and phases of implementation; practical considerations for selecting a type of hybrid study; and sampling considerations by implementation phase.

## Data Availability

No new data were generated or analysed in support of this research.
